# Effect of Statins on Lung Cancer Molecular Pathways: A Possible Therapeutic Role

**DOI:** 10.3390/ph15050589

**Published:** 2022-05-10

**Authors:** Gianmarco Marcianò, Caterina Palleria, Alessandro Casarella, Vincenzo Rania, Emanuele Basile, Luca Catarisano, Cristina Vocca, Luigi Bianco, Corrado Pelaia, Erika Cione, Bruno D’Agostino, Rita Citraro, Giovambattista De Sarro, Luca Gallelli

**Affiliations:** 1 Department of Health Science, School of Medicine, University of Catanzaro, 88100 Catanzaro, Italy; gianmarco.marciano@libero.it (G.M.); al.cas1993@gmail.com (A.C.); raniavincenzo1@gmail.com (V.R.); emanuele.basile1082@virgilio.it (E.B.); lucacatarisano@gmail.com (L.C.); cristina_vocca@live.it (C.V.); citraro@unicz.it (R.C.); desarro@unicz.it (G.D.S.); 2 Operative Unit of Clinical Pharmacology and Pharmacovigilanze, Mater Domini Hospital, 88100 Catanzaro, Italy; palleria@unicz.it (C.P.); luigibianco1969@gmail.com (L.B.); pelaia.corrado@gmail.com (C.P.); 3 Department of Pharmacy, Health and Nutritional Sciences, University of Calabria, Ed. Polifunzionale, Arcavacata di Rende, 87036 Rende, Italy; erika.cione@unical.it; 4 Department of Experimental Medicine L. Donatelli, Section of Pharmacology, School of Medicine, University of Campania Luigi Vanvitelli, 80100 Naples, Italy; bruno.dagostino@unicampania.it; 5 Research Centre FAS@UMG, Department of Health Science, School of Medicine, University of Catanzaro, 88100 Catanzaro, Italy

**Keywords:** statin, lung cancer, treatment, targets

## Abstract

Lung cancer is a common neoplasm, usually treated through chemotherapy, radiotherapy and/or surgery. Both clinical and experimental studies on cancer cells suggest that some drugs (e.g., statins) have the potential to improve the prognosis of cancer. In fact, statins blocking the enzyme “hydroxy-3-methylglutaryl-coenzyme A reductase” exert pleiotropic effects on different genes involved in the pathogenesis of lung cancer. In this narrative review, we presented the experimental and clinical studies that evaluated the effects of statins on lung cancer and described data on the effectiveness and safety of these compounds. We also evaluated gender differences in the treatment of lung cancer to understand the possibility of personalized therapy based on the modulation of the mevalonate pathway. In conclusion, according to the literature data, statins exert multiple effects on lung cancer cells, even if the evidence for their use in clinical practice is lacking.

## 1. Introduction

Lung cancer is the leading cause of neoplastic death worldwide and can be divided in two main types: NSCLC and SCLC. Over 80% of lung cancer cases belong to the NSCLC type, and can be divided into adenocarcinoma (40% of all NSCLCs), squamous cell carcinoma and large cell carcinoma [1,2]. Possible risk factors or causes include smoking, occupational chemicals, radon, genetic factors and lung diseases [3]. Lung cancer is characterized by a complex pathological expression of different molecular pathways. The analysis of SCLC is verified by the bi-allelic inactivation of RB1 and TP53 as obligatory mutations. RB1 function may be also compromised by an increased expression of cyclin D1. NOTCH inactivation, the kinase gene and TP73 mutations may also be part of the pathogenetic process [4]. MYC may exert an important evolutive action in SCLC [5]. MAPK and MEK5/ERK5 are also involved in cell proliferation and lipid metabolism, including the mevalonate pathway [6].

The metastatic potential of NSCLC correlates with the cellular expression of MMPs, which are regulated by NF-κB, by the metastasis suppressor RECK [7], as well as by the innate immune system.

Moreover, EGFR, ALK, ROS1, PDGFR, TP53, SOX2 and KRAS are involved in NSCLC pathogenesis and in its dissemination [8].

Lipid metabolism is related to cancer etiology and growth. Lipogenesis has a correlation with neoplastic aggressivity in certain neoplasms. The activation of the growth mechanism by neoplastic cells can lead to the production of unsaturated fatty acids, and certain metabolic enzymes or proteins are overexpressed in neoplastic cells (e.g., ACLY and FAS) [9]. In addition, the fragility of the blood vessels in cancer cells results in increased hypoxia and adipogenesis, which play a role in the signaling pathway and the production of the membrane structure [9,10]. Moreover, Kucharska-Newton et al. [11], studying 14,547 members of the ARIC cohort study during a 13-year follow-up, described an inverse association between plasma HDL cholesterol levels and lung cancer insurgence related to smoking status. In particular, the authors observed this association in former smokers (hazard ratio: 1.77), but not in current smokers. Other studies suggest that high cholesterol levels can negatively affect the clinical outcomes of cancer [9,12,13,14].

Although many compounds are used in the management of lung cancer, some authors have described other compounds acting on the cholesterol pathway that may be used. Statins are the most commonly used medications to manage hypercholesterolemia/dyslipidemia through the inhibition of HMG-CoA reductase [15] (Table 1 and Table 2).

Statins have pleiotropic effects, exhibiting beneficial effects (or even side effects) regardless of their action on cholesterol levels. For example, statins block the formation of isoprenoid intermediates, the prenylation of important binding proteins such as Rac and Rho and of their effectors. This can modulate nitric oxide synthase, platelets, reactive oxygen species, atherosclerotic plaque and cardiac remodeling [17]. The potential benefits are also documented in the nervous system: preventing strokes, modulating the immune response in multiple sclerosis and reducing the rate of insurrectional dementia. Statins seem to display an anti-inflammatory effect through NFκB, adhesion molecules and NOS modulation [18]. Experimental and epidemiological studies have described the antitumor properties of statins, speculating on their potential use in cancer therapy [14,19,20,21]. In this narrative review, we assessed the key evidence on the efficacy of statins in lung cancer and the possible molecular mechanisms involved.

## 2. Material and Methods

The PubMed, Embase and Cochrane library databases were searched for articles published until 28 February 2022 in the English language, in agreement with our recent papers [22,23,24].

The secondary search included articles cited in the reference lists of papers identified by the primary search. The records were first screened by title/abstract before full-text articles were retrieved for eligibility evaluation. The remaining articles were then subject to a citation search of all reference lists. Papers were deemed eligible if they included any of the words “statins”, “cholesterol pathway”, “lung cancer”, “pathway”, “molecular”, “effects”. All citations were downloaded into Mendeley and duplicates deleted. GM and CP screened all articles by title/abstract to determine their eligibility and CV reviewed a random sample of 20% to evaluate the reliability of the selection process. To avoid a bias of exclusion, the full text articles were retrieved following the first-round exclusions and were also subject to two independent eligibility reviews (CP 100%, VR 100%), this time with perfect agreement. The studies evaluated as eligible were included in the present review. We excluded manuscripts without full text and without indications of effects on cancer, and manuscripts not in the English language. AC revised the manuscript, explaining both anticancer mechanisms and pharmacokinetics properties.

## 3. Effects of Statins on Lung Cancer Molecular Pathways

Statins exert their effects on multiple pathways/proteins. These actions determine the antiproliferative, anti-angiogenic and pro-apoptotic effects. In addition, they may inhibit the expression, invasion and metastasis of cancer stem cells, although these proteins do not play a role in lung cancer [25].

### 3.1. Antiproliferative and Pro-Apoptotic Effects

#### 3.1.1. In Vitro and Preclinical Studies

Simvastatin is considered to enhance apoptosis, promote p53 mutant degradation and regulate lipid membranes [26,27,28]. In fact, Liu et al. [29] documented in their experimental studies that simvastatin reduces cell proliferation and the osteolytic bone metastasis of lung cancer cells, inhibiting the kinase phosphorylation in the MAPK/ERK signaling pathway.

In agreement with these data, in human cell culture, we documented that simvastatin significantly (*p* < 0.01) inhibited the proliferative effect of H_2_O_2_ (0.5 mM) and its stimulatory actions on ERK1/2 phosphorylation, NF-κB activation and IL-8 production. Furthermore, simvastatin decreased the H_2_O_2_-mediated induction of the cellular expression of MMP-2 and MMP-9, as well as of several components of the signaling complex activated by innate immune responses. Therefore, we suggest that simvastatin plays a role in antiproliferative, pro-apoptotic and anti-inflammatory effects in the presence of pro-inflammatory mediators [19].

In an in vitro study, Otahal et al. [30] evaluated the effects of pitavastatin and fluvastatin alone or plus erlotinib on the activation of different cell death pathways in three NSCLC cell lines. The authors documented that pitavastatin and fluvastatin induced cell death in EGFR TKI-resistant NSCLC cells lines, and these effects increased synergistically when pitavastatin was administered with erlotinib. The concomitant administration of mevalonic acid or caspase inhibitors restored cell status, suggesting that the mevalonate pathway is involved in these apoptotic effects of statins.

A pro-oncogenic role of p27 has recently been supposed. Podmiserg et al. [31] suggested that cytoplasmic p27 binds and inhibits the small GTPase RhoB, favoring its loss of function often associated with NSCLC. Moreover, lovastatin has inhibitory effects on both p21 and p27, sensitizing lung cancer cells to ionizing radiation [32].

In an experimental study, Zhang et al. [33] showed that the regulation of the BCL2/BAX/caspase-3 pathway by methionine enkephalin induced the apoptosis of lung cancer cells. Therefore, the statin-mediated activation of apoptosis may be useful in blocking neoplastic spread [25,34]. The effect of simvastatin on NFκB was associated with the derepression of PTEN and reduction of Bcl-xL (anti-apoptotic factor) in breast cancer [35].

**Figure 1:**
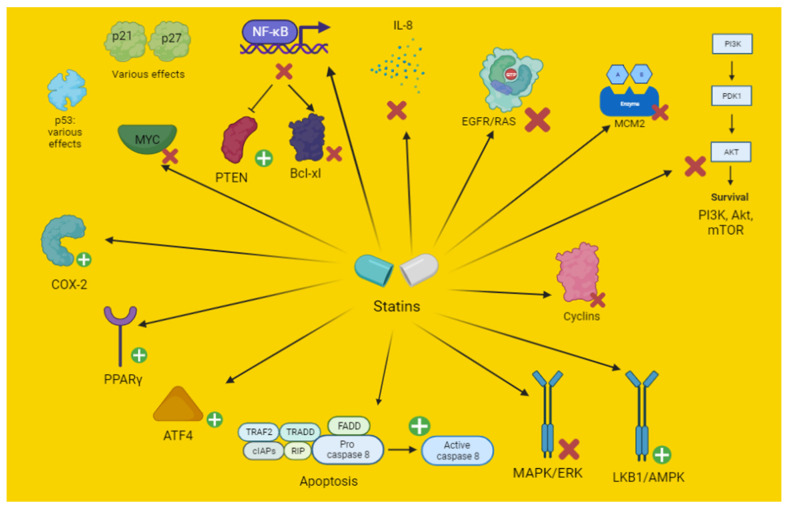
Schematic representation of the effect of statins on the mechanisms of apoptosis. Akt, protein kinase B; COX, cyclooxygenase; AMPK, 5′ adenosine monophosphate-activated protein kinase; ATF, activation of transcription factor; EGFR, epithelial growth factor receptor; ERK, extracellular signal-regulated kinases; FADD, Fas-associated protein with death domain; IAP, inhibitor of apoptosis; IL, interleukin; LKB1, liver kinase B1; MAPK, mitogen-activated protein kinase; MCM, minichromosome maintenance; mTOR, mechanistic target of rapamycin; MYC, myelocytomatosis (similar oncogene); NFκB, nuclear factor kappa B; PI3K, phosphoinositide 3-kinases; PPAR-γ, peroxisome proliferator-activated receptors-gamma; PTEN, phosphatase and tensin homolog; RIP, receptor interacting protein; TRADD, tumor necrosis factor receptor type 1-associated death domain protein; TRAF, TNF-receptor associated factors.

AMPK has an improved function in conditions of hypoxia or nutrient deprivation (low cellular ATP) and is overactivated in several cancer forms. AMPK has an inhibitory activity on the mevalonate pathway [25]. Conversely, it is of significant importance in KRAS-dependent NSCLC growth induced in murine models [36]. Statins have been shown to modulate AMPK function [25,32,37,38] (Figure 1) (Table 3 and Table 4). Simvastatin activates AMPK and modulates AMPK/Akt/mTOR signaling, reducing the oncogenic transformation through metabolic modulation. Atorvastatin and simvastatin induce cancer cell autophagy in gastrointestinal and glioma cancer, leading to increased cell survival through AMPK stimulation (Figure 1) [25,37]. Therefore, the co-administration of an autophagy inhibitor may result in an autophagy block and an increased sensitivity to statin-induced apoptosis [25,37,38]. Lovastatin increases the sensibility of lung cancer cells to irradiation and to apoptosis, mainly through AMPK stimulation and Akt inhibition [32].

RAS activation is subordinated to the binding of non-sterol isoprenoids synthetized by the mevalonate pathway. HMG-CoA reductase inhibition blocks the production of these compounds, acting on RAS. In fact, simvastatin and rosuvastatin seem to block SCLC line growth, inhibiting this target and reducing its expression in an in vitro study on SCLC patient samples [21,39].

Lovastatin showed in vitro pro-apoptotic action in lung cancer cells upregulating COX-2 and PPAR-γ [40].

#### 3.1.2. Clinical Studies

In a retrospective study, Chou et al. [26] showed a five-year mortality reduction in patients with TP53 mutated early-stage lung adenocarcinoma in treatment with simvastatin.

Statins appear to be effective in tumors, expressing MYC (these data are based on action on the MAPK/ERK pathway) and also inhibiting the PI3K pathway. The addition of high intensity statins (e.g., atorvastatin) to PD-1 inhibitors (i.e., nivolumab, pembrolizumab) resulted in an increased efficacy in mesothelioma and NSCLC. In an observational study, Cantini et al. [41] treated 253 patients with malignant pleural mesothelioma or advanced NSCLC with PD-1 inhibitors; the previous use of statins was associated with a better response and progression-free survival. These effects are expected to enhance the immune response against cancer cells, causing long-term retention of antigens on the cell membrane and feeding their presentation.

The importance of p21/p27 in the pathogenesis of lung cancer is controversial, as p21 was considered a positive prognostic marker since it contributes to cell cycle regulation. However, data on this pathway are not conclusive. Shoji et al. [42] evaluated the expression of p53 and p21 in 233 stage I–IIIA NSCLC patients. A total of 120 patients (51.5%) expressed p21, showing a better five-year survival rate (73.8%) compared to negative patients (60.7, *p* = 0.006). Patients who were p21 positive (wild type)/p53 negative had a greater five-year survival rate compared to patients who were p21negative/p53 positive (aberrant mutation). In a case-control study evaluating the frequencies of p21 polymorphisms in 27 healthy Koreans, Choi et al. [43] documented that both −2266 A and ht2-4 alleles were protective alleles, related to a significantly decreased risk of lung cancer.

In NSCLC patients, Xie et al. [44] documented that smoke use induces an overexpression of p53 or p21 that is associated with poor prognosis.

Protein p27 has an unclear role in lung cancer: patients affected by NSCLC have lower levels of p27 and PTEN. Protein p27 expression seems to be associated with a better prognosis [45]. In a systematic review, Zhuang et al. [46] reported that in NSCLC patients, a high p27 expression was associated with better survival. This data may be interesting if integrated with those reported in the previous section.

**Table 3:** Statins’ antiproliferative and pro-apoptotic effects in lung cancer.

Antiproliferative and Pro-Apoptotic Effects		
*In Vitro*		
**Statin**	**Dosage**	**Mechanism and Effects**
*Fluvastatin*	EGFR TKI-resistant NSCLC cell lines A549 and Calu6 (in combination with erlotinib). Minor effect in H1993 cells. 50–100 μM	Cells’ resistance related to K-RAS, EGFR or MET mutation. Inhibition of EGFR/K-RAS and then of Akt [30]
*Lovastatin*	0–50 μM	A549 lung adenocarcinoma cells were treated with lovastatin alone or in combination with 0 to 8 Gy IR. Lovastatin reduced EGF-induced phosphorylation of EGFR and Akt, and IR-induced Akt phosphorylation. Furthermore, lovastatin enhanced AMPK expression, and reduced p53 and the cyclin-dependent kinase inhibitors p21^cip1^ and p27^kip1 expression^ [32]
	Study conducted on A549 and H358 lung carcinoma cells Effect on viability: 76.7 μM (A549) and 45.2 μM (H358). Apoptotic effect: 50 μM (A549) and 75 μM (H358).	Pro-apoptotic action in lung cancer cells upregulating COX-2 and PPAR-γ [40]
	Two NSCLC cell lines, A549 and GLC-82: various concentrations (0, 2.5, 5, 10 and 20 μmol/L) for 1 to 4 days, respectively	MCM 2 is targeted by lovastatin in NSCLC cells. This inhibition led to cellular cycle block, inhibiting cyclin D1, CDK4 and Rb, but increasing p21 and p53 expression [47].
*Pitavastatin*	EGFR TKI-resistant NSCLC cell lines A549 and Calu6 (in combination with erlotinib). Minor effect in H1993 cells 50–100 μM. Improved effect with erlotinib 5 μM, with a pitavastatin dosage of 1–50 μM (Calu6) and 1–10 μM (A549).	Cells’ resistance related to K-RAS, EGFR or MET mutation. Inhibition of EGFR/K-RAS and then of Akt, resulting in increased apoptosis [30]
*Rosuvastatin*	1.25–30 μM	Reduction of RAS expression in an in vitro study on SCLC patients samples [21]
*Simvastatin*	28 μM in Bm7 (R248W) p53 mutant cells (cytotoxicity). 7 μM (IC_50_) in Bm7 cells (cell growth inhibition). 10 or 50 μM increased apoptosis in a dose-dependent manner in Bm7 cells. 1 μM decreased lipid raft and cell motility.	Cytotoxic, apoptotic, effect in p53 mutated cells. Cell growth, motility and lipid rafts inhibition, alongside mutant p53 degradation [26]
	2.5–30 μM (30 μM best results)	Reduction of RAS expression in an in vitro study of SCLC patient samples [21]
	Human lung cancer cell line A549: 10 and 50 μM	Decreased Bcl-2, cyclin D1 and CDKs, Xiap, NF-kB; increased Bax, caspase-3, -8, and -9 mRNA [34].
	H1975 NSCLC cells 2 μM for 48 h.	Expression of pro-apoptotic proteins was increased. Reduction of ERK 1/2 phosphorylation. Expression of BIM was blocked by gefitinib (1 μM), but significantly enhanced by simvastatin [48].
	GLC-82 human lung adenocarcinoma cell line: 30 μM	Inhibition of stimulatory actions on ERK1/2 phosphorylation, NF-κB activation and IL-8 production. Simvastatin decreased H_2_O_2_-mediated induction of the cellular expression of MMP-2 and MMP-9, as well as of several components of the signaling complex activated by innate immune responses, including MyD88, TRAF2, TRAF6 and TRADD [19].
*In vivo (animals)*		
*Simvastatin*	5–10 mg/kg in Balb/C nude mice A459 cancer cells	Inhibition of MAPK/ERK pathway. Increase of p53 expression [29].
*In vivo (humans)*		
*All statins*	Dosage commonly used for hypercholesterolemia	Action on P53, improved prognosis in early stage patients (10,975 patients analyzed retrospectively) [26]
*Atorvastatin*	Atorvastatin (40–80 mg). Observational study performed in 253 patients with malignant pleural mesothelioma or advanced NSCLC treated with PD-1 inhibitors.	Better response and progression-free survival. These effects probably due to immune enhancement related to a prolonged retention of antigens on cell membrane and presentation increase [41].
*Rosuvastatin*	Rosuvastatin (20–40 mg): high intensity. Observational study performed in 253 patients with malignant pleural mesothelioma or advanced NSCLC treated with PD-1 inhibitors.	Better response and progression-free survival. These effects probably due to immune enhancement related to prolonged retention of antigens on cell membrane and presentation increase [41].

**Table 4:** Statins’ possible antiproliferative and pro-apoptotic effects (further studies needed).

Antiproliferative and Pro-Apoptotic Effects		
*In Vitro*		
**Statin**	**Dosage**	**Mechanism and Effects**
*Lovastatin*	Mammary tumor cells: 0.25 μM	Combination low-dose statin and γ-tocotrienol induced cell cycle arrest at G1. Increased p27 and corresponding decrease in cyclin D1, CDK2, and hypophosphorylation of Rb protein [49]
	HGT-1 gastric cancer cells: 12.5 μM alone or in combination with docetaxel. Lovastatin tested alone: 2.5, 5 μM for 48–72 h.	Apoptosis increase (better in combination). Increase of p21 and p27, with reduction of aurora kinases A and B, cyclins B1 and D1. Cleavage of procaspase-3, reduction of Mcl-1 protein, Poly-ADP-Ribose Polymerase and Bax. HGT-1 cell derivatives overexpressing the MDR-1 gene were more sensitive to lovastatin than docetaxel-sensitive cells [50].
	10–50 μM in HNSCC cells	Activation of integrated stress response through ATF4 stimulation [51]
	1–25 μM SCC cells	Induction of LKB1 and AMPK activation [52]
*Mevastatin*	Mammary tumor cells: 0.25 μM	Combination low-dose statin and γ-tocotrienol induced cell cycle arrest at G1. Increased p27 and corresponding decrease in cyclin D1, CDK2, and hypophosphorylation of Rb protein [49]
*Pravastatin*	Mammary tumor cells: 10 μM	Combination low-dose statin and γ-tocotrienol induced cell cycle arrest at G1. Increased p27 and corresponding decrease in cyclin D1, CDK2, and hypophosphorylation of Rb protein [49]
*Simvastatin*	Mammary tumor cells: 0.25 μM	Combination low-dose statin and γ-tocotrienol induced cell cycle arrest at G1. Increased p27 and corresponding decrease in cyclin D1, CDK2, and hypophosphorylation of Rb protein [49]
	U251 and C6 glioma cell lines: 6 μM	AMPK, Raptor activation Downregulation of Akt, mTOR, p70 S6 kinase 1. These actions result in increased autophagy and cell survival reverted by autophagy inhibitors [53]
*In vivo (animals)*		
*Atorvastatin*	100 mg/kg three times a week in transgenic mouse (HCC model)	The inhibition of HMG-CoA reductase suppresses MYC phosphorylation through Rac GTPase [54]
	In vivo: 50 mg/kg/die for 21 days in BALB/c nude mice injected with HCC Huh7 cells. In vitro: CRC cells (HCT116), HCC cells (Huh7): 50 μmol/L atorvastatin for 2 and 5 days, respectively	Activation of AMPK, p21, promoting cell survival. A combination with an autophagy inhibitor may revert this effect [38].
*Lovastatin*	Daoy or D283 medulloblastoma cells: 10 and 40 μM Transfected mice: 1.0 mg/kg three times per week for 4 weeks	MYC inhibition through miR-33b increase [55]
*Simvastatin*	MDAMB-231 human breast cancer cell xenografts in mice: 5 mg/kg/die for 7 days	Inhibition of NFκB was associated with PTEN derepression and Bcl-xL reduction in breast cancer [35]

### 3.2. Chemotaxis, Invasion and Angiogenesis

#### 3.2.1. Experimental Studies

**Figure 2:**
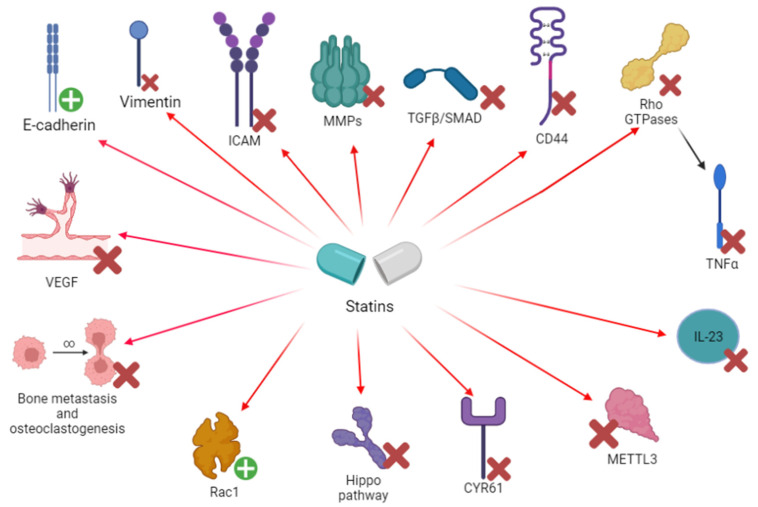
Schematic representation of the effects of statins on both protein (and receptors) and cells involved in angiogenesis and metastasis. CD, cluster of differentiation; CYR61, cysteine-rich angiogenic inducer 61; GTP, guanosine triphosphate; ICAM, intercellular adhesion molecule; IL, interleukin; METTL3, methyltransferase 3; MMP, metalloproteinase; Rac, ras-related C3 botulinum toxin substrate; Rho, ras homologous protein; SMAD: small mother against decapentaplegic; TGF, transforming growth factor; TNF, tumor necrosis factor; VEGF: vascular endothelial growth factor.

The enhancement of epithelial markers (e.g., E-cadherin) and the reduction of mesenchymal markers (vimentin) may exert a role in the neoplastic process. In fact, EMT is an important process leading to invasion and metastasis [56,57]. An in vitro experiment on A459 lung cancer cells by Yang et al. showed that simvastatin reduced TGF β1-dependent EMT, preventing E-cadherin decrease and reducing the levels of three EMT markers: α-SMA, Vi and FN [56]. According to Nishikawa et al., the in vitro effects of statins in reducing EMT and invasion seem to be stronger in p53 mutated cells from patient specimens [58] (Figure 2) (Table 5 and Table 6).

In an in vitro (A459 cells)/in vivo (murine model) study by Liu et al. [29], simvastatin determined a reduction of CD44, MMP2 and MMP9 (*p* < 0.01). Simvastatin inhibited tumor growth and invasion through MAPK/ERK inhibition. Simvastatin and rosuvastatin reduced MMP2 and MMP9 (*p* < 0.01) expression in lung cancer tissue samples collected from 12 patients undergoing surgery; MMPs concentration is regulated by NFκB and its expression is also reduced by statins in normal lung tissue, whereas action on MMPs only occurred in cancer tissue [21] (Figure 2) (Table 5 and Table 6).

The Rho GTPase family plays a role in cancer cells’ adhesion and migration [59]. Statins (cerivastatin) showed a certain effect in modulating/inhibiting these proteins in breast and prostate cancers [25,60,61] (Table 5 and Table 6). Lung cancer has a various degree of dependence by Rho GTPases activity and Rho modulators actions [62,63,64,65], and the relevance of statins’ action on this protein in lung cancer needs to be further understood. It is not futile to remember that Rho activity is modulated by products from the mevalonate pathway [25].

E-selectin is an adhesion protein involved in cell migration. Lovastatin was effective in reducing E-selectin levels through the Rho-mediated inhibition of TNFα [66].

CYR61 takes part in chemotaxis, cell migration and adhesion, cell survival, angiogenesis and DNA synthesis. Its role in lung cancer is controversial. Some authors described reduced CYR61 mRNA levels in cancer cells and its overexpression seemed to reduce neoplastic spread and growth. However, other papers reported its effects in cell growth, EMT, cell viability and migration enhancement. Some papers discussed the correlation between CYR61 and lung cancer, but without conclusive results [67,68]. Statins (i.e., atorvastatin, simvastatin, cerivastatin and pravastatin) have been shown to act on CYR61 in osteosarcoma cells (Table 5 and Table 6) [69].

Cerivastatin was effective in inhibiting the Hippo pathway, acting on YAP/TAZ and reducing the expression of YAP-targeted oncogenes (EGFR, AXL, CYR61 and TGFbR2). This mechanism was effective in increasing ALK inhibitors’ effects in resistant tumor cells in both in vitro and in vivo murine models, created by Yun and colleagues [70] (Figure 2) (Table 5 and Table 6).

In experimental models, Fluvastatin inhibited lung cancer bone metastasis and invasion [71,72], while simvastatin reduced osteoclastogenesis, through RANK-L and IL-6 inhibition [73] (Table 5 and Table 6).

#### 3.2.2. Clinical Evidence

VEGF is involved in cancer spread, vascularization and metastasis through its action on the endothelium in NSCLC patients. Moreover, VEGF action is regulated by Rho GTPase and statins act on both targets [74]. Weis et al. [74] documented that cerivastatin and atorvastatin showed a modulatory effect on angiogenesis: low-dose statin was associated with proangiogenic properties, whereas a high dose generated an antiangiogenetic effect. A meta-analysis in the general population observed a relevant reduction of VEGF in patients treated with statins [75] (Table 5 and Table 6).

E-selectin is highly expressed in NSCLC patients. Ozmen and Simsek [76] reported high levels of IL-23, ICAM and E-selectin in patients with NSCLC that decreased after radiotherapy. A similar effect was described in patients after statin treatment (especially simvastatin). In particular, in a review of the literature, Zinellu and Mangoni [77] found that statins are able to decrease the plasma levels of E-selectin and P-selectin.

High CYR61 levels seem to be associated with a less aggressive disease in lung cancer and may be a possible biomarker of this neoplasm in men. However, the data were acquired on few patients [78,79].

**Table 5:** Statins’ effects on chemotaxis, invasion and angiogenesis in lung cancer.

***In Vitro***		
*Rosuvastatin*	Lung cancer patient samples: 1.25–30 μM	Simvastatin reduced MMP2 and MMP9 (*p* < 0.01) expression in lung cancer tissue. Reduction of NFκB, both in normal and cancer tissue [21]
*Simvastatin*	10–20 μg in A459 cells [56] 5–10 mg, analyzed in human samples (H1650 and H1975 cells) [58]	Inhibition of EMT, inhibiting TGF-β1 activity and SMAD pathway [56] 250 specimens from lung cancer patients, 51 of whom were treated with statins (mainly simvastatin), revealed an improved prognosis [58]
	Lung cancer patient samples: 2.5–30 μM	Simvastatin reduced MMP2 and MMP9 (*p* < 0.01) expression in lung cancer tissue. Reduction of NFκB, both in normal and cancer tissue [21]
	A459 cells: 0, 5, 10 or 20 μM (the latter being the main dose) for 24 h.	METTL3 inhibition, thus reducing EZH2 activity in generating EMT in lung cancer cells. METTL3 may regulate the levels of EMT-associated genes, including JUNB [80].
	Human lung cancer cell line A549: 10 and 50 μM	MMP-9 suppression [34]
	5–10 mg/kg in Balb/C nude mice A459 cancer cells	Reduction of CD44, MMP-2 and MMP-9 [29]. Proliferation and bone metastasis inhibition
***In vivo (animals)***		
*Atorvastatin*	10 mg/kg per day in mice	VEGF inhibition, through the blocking of ROS production, the suppression of Rac1/NADPH oxidase activity and the upregulation of glutathione peroxidase and catalase [81]
	Endothelial cells: 0.005 to 0.01 μmol/L dose led to increased vascularization 0.05–1 μmol/L dose led to decreased vascularization Mice: In lung cancer murine models, inflammation-induced angiogenesis was enhanced with low-dose statin therapy (0.5 mg/kg/die), reduced with high dose (2.5 mg/kg/die)	Decreased VEGF at high dose [74]
*Cerivastatin*	Endothelial cells: 0.005 to 0.01 μmol/L dose led to increased vascularization 0.05–1 μmol/L dose led to decreased vascularization Mice: In murine models, inflammation-induced angiogenesis was enhanced with low-dose statin therapy (0.5 mg/kg/die), reduced with high dose (2.5 mg/kg/die)	Decreased VEGF at high dose [74]
	In vitro and in vivo murine models (1 mg/kg daily)	Cerivastatin was effective in inhibiting Hippo pathway, acting on YAP/TAZ and reducing expression of YAP-targeted oncogenes (EGFR, AXL, CYR61, and TGFbR2) [70]
*Fluvastatin*	Mouse model: fluvastatin, 50 mg/kg	Fluvastatin may inhibit lung cancer bone metastasis [71]. Fluvastatin induced cell autophagy, preventing their spread (related to p53 increase) [72].
*Simvastatin*	Inoculation of A459 cells in mice: 10 mg/kg simvastatin every day for 7 days	Reduction of CD44, MMP2 and MMP9 [29]

**Table 6:** Statins’ actions on chemotaxis, invasion and angiogenesis.

Chemotaxis and Invasion		
*In Vitro*		
*Atorvastatin*	In osteosarcoma cells: 10 μM	CYR61 silencing was an unfavorable setting for cancer proliferation, resulting in increased cell death [69]
*Cerivastatin*	25 ng/mL in breast cancer MDA-MB-231 cells [60]. Aggressive breast cancer cell line, characterized by RAS and NFκB overactivation and RhoA high expression	Target: Rho GTPases (inhibition) Rho A inhibition may also result in NFκB and MMP downregulation
	In osteosarcoma cells: dosage not available	CYR61 silencing was an unfavorable setting for cancer proliferation, resulting in increased cell death [69]
*Lovastatin*	0.1–1 μM in a study on colon carcinoma cells. Inhibitory concentration of lovastatin was in a physiologically relevant range (IC50 < 0.1 μM)	Lovastatin was effective in reducing E-selectin levels through Rho-mediated inhibition of TNFα [66]
*Pravastatin*	In osteosarcoma cells: dosage not available	CYR61 silencing was an unfavorable setting for cancer proliferation, resulting in increased cell death [69]
*Simvastatin*	HCT116 colorectal cancer cells: 10 μg	Paradoxical activation of RhoA, Cdc 42 and Rac1. Statins may also paradoxically activate Rho GTPases and their action is not easy to predict. Rho GDIα inhibition removed [61]
	In seven human/murine osteosarcoma cells: dosage not available	CYR61 silencing was an unfavorable setting for cancer proliferation, resulting in increased cell death [69]
*In vivo (animals)*		
*Simvastatin*	In a mouse model of bone loss: 10 mg/kg Mouse derived macrophage-like cells were observed and mice bone mineral density	Simvastatin reduced osteoclastogenesis, a process enhanced by neoplastic cells, through RANK-L and IL-6 inhibition. Probable IRF4, NFκB and NFATc1 inhibition [73]
*In vivo (humans)*		
*All statins (mainly lipophilic statins)*	Hypercholesterolemia dosage	Relevant reduction of VEGF in patients treated with statins. This effect was observed in patients treated with lipophilic statins, therapy duration ≥ 4 weeks, LDL-C reductions ≥ 50 mg/dL, and among people affected by a relevant comorbidity (in general population) [75]
	Hypercholesterolemia dosage, various dosages	E-selectin and P-selectin reduction: statins (especially simvastatin) [77]
*Atorvastatin and Rosuvastatin*	184 aneurysmatic patients (two groups: statin and non-statin). Group III: non-aneurysmatic patients in statin treatment. ≤40 mg rosuvastatin, ≤80 mg atorvastatin	MMPs and NGAL reduction [39]

##### Cancer Stem Cells

**Figure 3:**
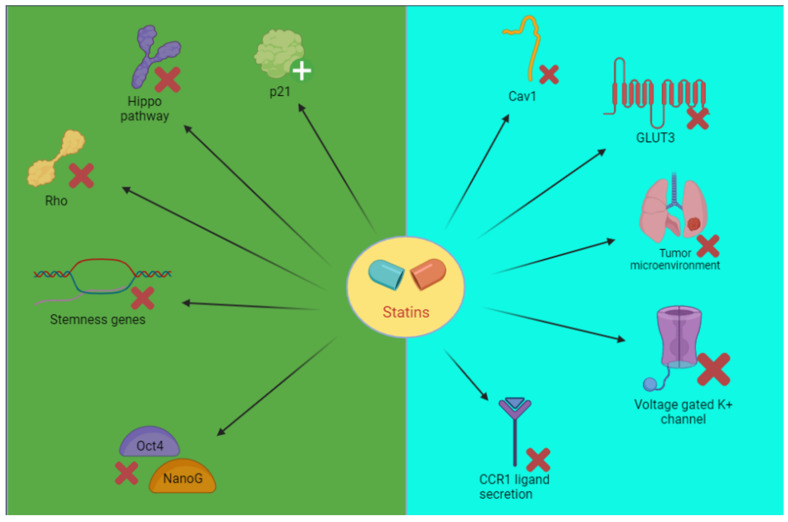
Schematic representation of the effects of statins on stem cells (green) or other possible mechanisms (light blue). Cav, caveolin; CCR, chemokine receptor type; GLUT, glucose transporter; Oct4, octamer-binding transcription factor 4; Rho, ras homologous protein.

Experimental studies documented that treatment with statins prevented the active substitution of stem cells by RhoA and the impairment of YAP/TAZ (reduced pro-oncogene effect) and then inhibited Oct4/Nano G [25]. Blocking Hippo pathway statins plays an important role in lung cancer. YAP/TAZ are involved in cell proliferation and migration. YAP/TAZ regulates many microRNAs in NSCLC and is involved in its etiology, inducing MCM7 activity and inhibiting p21 [82] (Figure 3) (Table 7 and Table 8).

##### Other Targets

Ali et al. [83] reported that atorvastatin has an in vitro/in vivo action against TKI-resistant NSCLC. It inhibits Cav1 that regulates malignant cell proliferation and GLUT 3. This axis has a role in TKI-resistant cells (Table 7 and Table 8).

Minichromosome maintenance 2 is targeted by lovastatin in NSCLC cells. This inhibition leads to the blocking of the cellular cycle (Table 7 and Table 8) [47].

In A459 lung cancer cells, Chen et al. [80] reported that simvastatin inhibits METTL3 (Table 7 and Table 8) [84]. Finally, statins have been shown to target the tumor microenvironment, reducing the crosstalk between primary cancer cells and mesenchymal cancer cells. However, the comprehension of the microenvironment and its therapeutic role is still incomplete [85,86] (see Table 7 and Table 8).

**Table 7:** Statins’ effects on lung cancer stem cells or other mechanisms.

Cancer Stem Cells		
*In vitro*		
*Cerivastatin*	1 μM in H1299 NSCLC cells	YAP/TAZ inhibition, MCM7 inhibition, p21 restoration [82]
**Other targets**		
*In vitro*		
*Simvastatin*	5 or 10 μM in human SCC cells. Action on microenvironment	Simvastatin inhibits the MSCs–PCCs crosstalk. Pleiotropic effects on cell metabolism, suppression of IL-6 and CCL2 production by MSCs and CCL3 secretion by PCCs [85]
*In vivo (animals)*		
*Atorvastatin*	In vitro/in vivo study: Mice: 30 mg/kg Cells (TKI-sensitive (PC-9 and HCC827) and resistant (PC-9GR, H1975, and H1703)): 0.2, 1 or 5 μM	Atorvastatin (in combination with tyrosine kinase inhibitors, TKI) showed a specific in vitro/in vivo action against TKI resistant NSCLC cells. Inhibition of Cav1 and GLUT 3 [83]
	Lung adenocarcinoma mice: 10 mg/kg/day	Inhibition of pro-tumorigenic macrophages in the tumor microenvironment. Inhibition of Rac-mediated CCR1 ligand secretion [86]

**Table 8:** Statins’ possible effects on cancer stem cells or other possible mechanisms (further studies needed).

Cancer Stem Cells		
*In Vitro*		
*Lovastatin*	Studied in hESC (HES3), karyotypically abnormal hESC (BG0IV), embryonal carcinoma (NTERA-2), ovarian (TOV-112D) and colorectal cancer (HT-29) cells 1−20 μmol/L (significant at 20 μmol/L)	Ineffective in hESC. However, BG01V, NTERA-2, TOV-112D and HT-29 were inhibited (apoptosis in karyotypically abnormal cancer cells, suppression of stemness-genes on chromosome 12 and 17) [87]
	Abnormal hESCs (BG01V) and breast adenocarcinoma cells (MCF-7)	Downregulation of Oct4 and NanoG, stemness gene reduction [88]
*Mevastatin*	Studied in hESC (HES3), karyotypically abnormal hESC (BG0IV), embryonal carcinoma (NTERA-2), ovarian (TOV-112D) and colorectal cancer (HT-29) cells 1−20 μmol/L (significant at 10 μmol/L)	Inhibition of cell proliferation [87]
	Abnormal hESCs (BG01V) and breast adenocarcinoma cells (MCF-7)	Downregulation of Oct4 and NanoG, stemness gene reduction [88]
*Simvastatin*	Mouse embryonic stem cells (J1, D3, and RW.4): 10 μM	RhoA and YAP/TAZ inhibition. Oct4 and NanoG downregulation [88,89]
	Abnormal hESCs (BG01V) and breast adenocarcinoma cells (MCF-7):5, 10, 20 mmol/L	Downregulation of Oct4 and NanoG, stemness genes reduction [88]
	Studied in hESC (HES3), karyotypically abnormal hESC (BG0IV), embryonal carcinoma (NTERA-2), ovarian (TOV-112D) and colorectal cancer (HT-29) cells 1−20 μmol/L (significant at 20 μmol/L)	Simvastatin was the most potent inhibitor. Ineffective in hESC. However, BG01V, NTERA-2, TOV-112D and HT-29 were inhibited (apoptosis in karyotypically abnormal cancer cells, suppression of stemness-genes on chromosome 12 and 17) [87]
**Other targets**		
*In vitro*		
*Mevastatin, pravastatin and simvastatin*	Neoplastic T cells Jurkat: 1.5 μM to 50 μM 30 μM (mevastatin and simvastatin), 50 μM (pravastatin) being the main dosages	Target: voltage gated potassium channel of the Kv1.3 type [84] Simvastatin was the more potent drug, pravastatin the less. Inhibitory effect was partially irreversible with simvastatin and completely reversible with pravastatin and mevastatin

## 4. Statins’ Effectiveness in Lung Cancer

A retrospective study by Cardwell et al. [90] described the prognosis improvement in patients consuming statins before or after diagnosis (Table 9).

In a retrospective study, Lin et al. [91] evaluated 5118 patients > 65 years of age and found improved survival in patients with NSCLC stage IV treated with statins. An efficacy correlation between the treatment duration/number of administrations was observed [92,93,94,95]. Leigh et al. [97] retrospectively evaluated the brain metastasis risk in 252 lung cancer patients. No significant results were described. In this study, 73 patients were treated with statins, and only 55 out of 252 developed brain metastasis. Therefore, no conclusions can be made.

We analyzed five clinical trials on statin effects in lung cancer [98,99,100,101,102] (Table 9). In a randomized phase II clinical trial in 106 patients with advanced-stage NSCLC, Han et al. [100] failed to report the superiority of gefitinib + simvastatin compared to gefitinib alone. However, the experimenters suggest that simvastatin may increase gefitinib’s effect in resistant NSCLC (Table 9).

Fiala et al. [98] reported better outcomes in 67 advanced NSCLC patients (holding KRAS mutation) treated with EGFR-TKI plus simvastatin/atorvastatin than EGFR-TKI alone. However, the authors did not declare the indication for statin administration, and this may generate bias.

Four metanalyses/systematic reviews substantially established the absence of significant effects from statins on lung cancer, based on existing trials. However, these papers confirmed some effect in all observational studies and the insufficiency of a congruent number of RCTs [103,104,105,106].

## 5. Gender Influence on Statins’ Effect in Lung Cancer

Sex specificity in lung cancer is a complex and trending topic. Several differences have been observed between males and females. Oncogenes p53 and KRAS mutations are more probable in women than in men. Smoking-associated damage is greater in women compared with men. The proliferation-stimulating peptide GRPR, HER-2 mutation and somatic EGFR mutation are more frequent in non-smoking women. DNA adduct levels showed superior quantities in women, increasing their probability for lung cancer [107,108]. Carcinogenic compounds present in smoke are variously metabolized. Among them, nitrosamines and, in particular, NNK have an important role in the pathology onset. The metabolic pathways of NNK embrace a chemical activation by CYP450 enzymes (e.g., CYP1A2, CYP2D6 and CYP3A4) and then conjugation with glucuronic acid. Estrogens seem to affect this process (since they are metabolized by the same CYP isoforms), reducing NNK activation. However, estrogens also inhibit NNK detoxication to its metabolite NNAL, and this interaction seems to be of major relevance, contributing to oncogenesis [108]. Women are expected to have higher lung CYP1A1 expression and a major level of detoxification enzymes polymorphisms (glutathione S-transferase), resulting in a higher carcinogen activation. A defective DNA repair system in women may also lead to increased toxicity in women [107].

Estrogen’s role in carcinogenicity is a controversial topic. Women in HRT or consuming contraceptives seem to have a greater incidence of lung cancer [108]. Most women are diagnosed in the pre-menopausal period, meaning that estrogens may have a certain correlation with neoplasm insurgence. Endogenous or exogen estrogens seem to have a certain grade of association with cancer onset, and even males receiving estrogens may show this evidence [108]. In an experimental study, treatment with fulvestrant (estrogen receptor antagonist) plus gefitinib (EGFR-TKI) reduced both cell proliferation and tumor volume [109]. In a mouse model of lung adenoma/adenocarcinoma [110], Hammoud et al. evaluated the role of estrogen in cancer. Baseline female mice showed higher tumor volume and count compared to males. Female mice had a reduced cancer volume and count after ovariectomy, and estrogen treatment increased lung cancer dimensions in male mice.

Differences in immune response have been reported between men and women. In general, immune system activity is estimated to be higher in women: some of the most important genes involved in immunity are located on X chromosome (e.g., TLR7, FOXP3), variations on white cells count/activity (e.g., higher CD4/CD8 ratio in women) are expected, and estrogen receptors alpha (more in T cells) and beta (more in B cells) have a differential expression in immune cells and may have a role in lung carcinogenesis. Androgens may inhibit NFκB, reducing inflammation. STAT3 deletion in mice with mutant KRAS led to carcinogenesis in male mice, whereas high-dose estrogens prevent this action in females. Nevertheless, estrogens reduce immune activity at high doses and increase it at low doses. Moreover, PD-L1, a ligand that favors tumor immune escape, may be regulated by estrogens.

Another important consideration is related to the major ability of female cancer cells to evade the immune response. However, further studies are needed. All of these considerations may affect immunotherapy [111], and possibly statins or mevalonate pathway-acting compounds in co-treatment.

The different immune response between genders is probably associated with a different prognosis in different lung cancer types. A better prognosis is generally expected in women’s NSCLC [112]. Immune checkpoint blockade therapy is under examination to observe potential treatment bias between men and women. Data from meta-analyses are not conclusive, and several confounding factors are present (including behaviors and lifestyle). Ye et al. observed a divergent pattern of sex-associated variability, especially in melanoma and lung cancer, including different immune checkpoints. Although no definitive conclusions can be made, this observation may be the model for further studies and trials [113]. The gender-related variability of statins’ efficacy and adverse events is another issue. Statins are recommended by guidelines in cardiovascular disease, but they have not been tested in women with the same evidence and numbers of men. A systematic review by Bandyopadhyay et al. [114] showed that a secondary prevention trial before 2001 comprised only 23% women and a primary prevention trial only 10%, limiting data effectiveness in this class. People aged > 75 years were also scarcely represented. A meta-analysis conducted by Dale et al. [115] found that statins are associated with reduced cardiovascular events in both women and men. In men, the authors documented a reduced rate of myocardial infarction, mortality and stroke, whereas in women, they did not evidence any benefit on these outcomes. However, several limitations affected this paper, including the lack of specific clinical trials. A pooled analysis of 22,231 patients showed little difference between men and women treated with statins or with statin + ezetimibe, assessing the necessity of gender-tailored statin use. The overall chances of achieving lower LDL (with men having better results in the statin + ezetimibe group) and ApoB levels were higher in women, whereas hs-CRP lowering, or combined hs-CRP/LDL lowering, was more probable in men [116].

The cardiovascular death rate in women is higher than in men, but the incidence of CVD is lesser until menopause [117]. Cardiovascular pleiotropic effects may have a higher relevance in women.

CYP3A4 could also play a role in gender differentiation. In fact, CYP3A4 is more active in women, and this may explain the reduced effect of statins that are metabolized by this cytochrome (e.g., simvastatin, atorvastatin) [117,118]. However, interactions between estrogens and statins are possible, since estrogens are metabolized by CYP450 enzymes, are conjugated with glucuronic acid and are also OATP1B1 substrates. However, in vitro studies evidenced CYP3A4/3A5 induction by estrogens, explaining the quicker statin metabolism in women [119].

## 6. Discussion and Conclusions

Statins have been shown to have preclinical effects in several in vitro/in vivo [21,25] and observational studies, but not in RCTs, often testifying a certain grade of benefit in lung cancer patients [103,104,105,106]. A series of important questions arise in the scientific community.

A combination of statins and different therapeutic agents has been proposed in this clinical setting. For example, sulindac, ABL allosteric inhibitors, SREBP-2 targeting and MEK-ERK 5 inhibition were potential good fits for statin coadministration [6,120,121,122]. In some trials, statins were proposed as an additive to standard chemotherapy [99,102].

New ways of administration may be evaluated for statins in lung cancer. In fact, inhalation could be a specific and interesting choice for these compounds. In this way, higher concentrations may reach the lungs using lower dosages [83,123].

The molecular heterogeneity of lung cancer is of key relevance in terms of its therapy. Physicians and experimenters should not only distinguish between different histotypes, but must consider other important factors. There are often significant genomic/proteomic variations between patients and sometimes in the same neoplasm, creating intertumor (diversity between tumor and metastasis), interpatient (genetic and phenotypic variations in the same tumor type) and intratumor (subclonal changes in the tumor cells) heterogeneity. Genetic, epigenetic and non-genetic mechanisms may all contribute to this scenario, influencing therapeutic response. Interestingly, the effects of simvastatin varied in murine models in different mutated p53 forms [28]. The microenvironment may also have a certain level of importance in this biologic process. Moreover, smoking seems to play an important role in the expression of the biomarkers involved in lung pathology and statin response [8,21]. In a phase II trial, smokers had a better response in outcomes after treatment with simvastatin + irinotecan + cisplatin compared to those who had never smoked. However, the cohort was too small to make definitive statements [101]. Similarly, there was a certain correlation between smoking, TNM staging, lymph node metastasis, differentiation and miRNA-21, PTEN and p27 expression [46].

The mevalonate pathway and lipid metabolism can influence some markers involved in cancer pathogenesis or the other way around. For example, there is a relationship between lipid metabolism variation and EGFR expression [9]. Further studies may be helpful to understand the reciprocal connection of these components and the action of statins on these pathways. This possible interaction was evaluated in a meta-analysis of case-control studies assessing the relationship between dietary cholesterol and lung cancer insurgence. The authors obtained inconsistent results and highlighted the need for further analysis [124].

Another important point is that the same gene/protein/mechanism may act variably as a tumor suppressor or enhancer in different contexts (such as Cav1) [83]. Autophagy can favor cancer cell survival, but can also prevent clinical metastasis [38,72]. Therefore, the effects of statins may be influenced by these variations.

Regarding statin effectiveness, it is important to consider that observational studies’ results describe a decrease in mortality. This conclusion could be biased by the statins’ decrease of cardiovascular risk, recall bias, and lack of stratification and/or history information. Concomitant treatment and staging were not assessed [91].

A study by Kamata et al. [86] showed that long-term treatment with atorvastatin led to drug resistance in mouse models. Inhibiting/avoiding this mechanism may be of crucial importance if statins are to show effects in clinical trials.

However, statins (i.e., atorvastatin, simvastatin, fluvastatin, lovastatin) may inhibit immune escape through PD-L1 decreasing in melanoma and lung cancer [125]. Furthermore, IDO inhibition (IDO1, in particular) may be an interesting target to be evaluated in statins’ actions. In fact, IDOs are involved in tumor resistance and are related to worse outcomes. Blocking this target may be extremely useful in cancer treatment, but further studies are needed [126].

Finding a biomarker able to predict the response to statins is another issue. Paula-Fernandez et al. [127] evaluated samples obtained from 90 NSCLC patients and documented that ACSL3 expression is related to a better simvastatin response, but a worse clinical outcome.

Chemotherapy may influence statin effects in certain clinical settings. Patients with liver metastasis may have higher statin blood levels due to hepatic damage. Chemotherapy may improve liver function, thus reducing the levels of statin in the blood. Physicians should be aware of this possibility [128].

Another crucial point is that statin-associated muscle symptoms may impact negatively on the quality of life of patients with lung cancer, who often also have sarcopenia, and may be a prevalent reason for the discontinuation of statin therapy in this population [129,130,131].

Although there is uncertainty regarding the real effects of statins in lung cancer, their properties may be the basis for producing new selective compounds that are active in the mevalonate pathway as adjuvant treatment in malignancies. Farnesyl transferase inhibitors, PTI, GGTI and FPPS inhibitors are under examination. Nevertheless, the toxicity of these compounds (especially GGTI) and the lack of sufficient advanced trials are major issues. Among other repurposed drugs, NBP showed activity on FPPS, probably due to their N-atom [25].

In conclusion, the role of statins in lung cancer is mainly based on experimental or retrospective/observational studies. There are few RCTs available, often contradicting the hypothesis of the effectiveness of this drug class. It is important to evaluate the chemical nature of single statins, patients’ genomics, intratumor differences, the microenvironment, histotypes and epigenetic factors to answer this question. RCTs should be performed using a single statin type on a specific NSCLC or SCLC histologic typology and mutations. Patients should be randomized while considering their previous exposure to smoke or other etiologic and epidemiological factors, as well as tumor characteristics.

## Figures and Tables

**Table 1:** Statins pharmacokinetics.

	BA [16]	t_1/2_	Hydrophile/Lipophile	Metabolism	Protein Binding
*Simvastatin*	<5%	2 h	Lipophile	Hepatic (first-pass, CYP3A4)	>95%
*Pravastatin*	17–18%	1.8 h	Hydrophile	Hepatic (first-pass)	50%
*Atorvastatin*	12%	14 h	Lipophile	Hepatic (CYP3A4)	≥98%
*Lovastatin*	≤5%	3 h	Lipophile	Hepatic (first-pass, CYP3A4)	≥95%
*Fluvastatin*	24%	1.2 h	Lipophile	Hepatic (CYP2C9)	≥98%
*Rosuvastatin*	20%	19 h	Hydrophile/lipophile	Scarcely metabolized (10% by liver: CYP2C9, with minor involvement of 2C19, 3A4 e 2D6 isoforms	~90%
*Pitavastatin*	51–80%	5.7–8.9 h	Lipophile	Mainly unmodified Liver has a minor role (UGT1A3 and 2B7; CYP2C8/9)	>99%

**Table 2:** Statins excretion and dose adjustment.

	Dosage	Elimination	Dose Adjustment Kidney Impairment	Dose Adjustment Hepatic Disease
*Simvastatin*	5–80 mg	13% urine 60% feces	eGFR < 30 mL/min: dosage superior to 10 mg/die should be evaluated carefully	Contraindicated in active hepatic disease or persistent transaminases increase
*Pravastatin*	10–40 mg	20% urine 70% feces	Moderate–severe kidney impairment: 10 mg starting dose, with follow-up	Contraindicated in active hepatic disease or persistent transaminases increase (3 times superior to upper limit) In some cases, 10 mg dosage is a possible option, with follow-up
*Atorvastatin*	10–80 mg	95% bile/feces <5% urine	No dose adjustment needed	Contraindicated in active hepatic disease or persistent transaminases increase (3 times superior to upper limit) Use carefully in other patients with hepatic impairment
*Lovastatin*	20–40 mg	83% feces 10% urine	Severe kidney impairment (eGFR ≤ 30 mL/min): doses superior to 20 mg must be evaluated carefully	Contraindicated in active hepatic disease, transaminases increase, cholestasis
*Fluvastatin*	20–80 mg	Major quote excreted in feces ≤6% urine	Mild–severe kidney impairment: no expected pharmacokinetics variation. However, carefully administer doses > 40 mg/die, in case of severe kidney impairment	Contraindicated in active hepatic disease, transaminases increase
*Rosuvastatin*	5–40 mg	90% feces (unmodified) 10% urine	Moderate kidney impairment (eGFR < 60 mL/min): starting dose 5 mg 40 mg dose is contraindicated Severe kidney impairment: do not administrate	Contraindicated in active hepatic disease, transaminases increase Child–Pugh 8–9: consider evaluation of kidney function Child–Pugh > 9: no data available
*Pitavastatin*	1–4 mg	95% feces (unmodified, enterohepatic circulation) 5% urine	No dose adjustment in patients with mild kidney impairment. However, caution is needed 4 mg dose is contraindicated in patients with severe kidney impairment	Contraindicated in active hepatic disease or persistent transaminases increase (3 times superior to upper limit) Child Pugh A and B: max 2 mg Child Pugh C: contraindicated

**Table 9:** Human studies of statins in lung cancer.

In Vivo (Humans)		
*Retrospective Studies*		
Statin	Dosage and Patients	Effectiveness
*All statins*	Dosage commonly used for hypercholesterolemia	Improved prognosis in early-stage patients (10,975 patients analyzed retrospectively) [26]
	NSCLC stage IV patients: hypercholesterolemia dosage	A cohort of 5118 patients was examined and the statin group had a better survival rate [91]
	3638 lung cancer patients (after diagnosis) 11,051 lung cancer patients (before diagnosis) Hypercholesterolemia dosage	Prognosis improvement in patients consuming statins before (better outcome) and after diagnosis. Lipophilic statins and patients with at least 12 prescriptions had better results [90]
	295,925 patients with 13 different cancer types, 18,721 used statins regularly before the cancer diagnosis: hypercholesterolemia dosage	Reduced cancer mortality [92]
	7280 patients receiving statins and affected by lung cancer in a larger court. Hypercholesterolemia dosage	Statin use > 6 months was associated with a risk reduction of lung cancer of 55% [93]
	5990 lung cancer patients in a larger cohort. Hypercholesterolemia dosage	Statins showed mortality reduction, especially in combination with metformin and aspirin [94]
	41 lung cancer patients (statin group) compared to 792 non-statin group. All patients treated with EGFR-TKIs. Hypercholesterolemia dosage	Better mortality in statin group, especially in tumors < 3 cm and with a CCI score < 3 [95]
	43,802 COPD patients: 10,086 used statins, whereas 33,716 did not Hypercholesterolemia dosage	Liu et al. retrospectively found that the risk of COPD evolution in lung cancer was reduced by statins [96]
*Atorvastatin*	Atorvastatin (40–80 mg) Observational study performed in 253 patients with malignant pleural mesothelioma or advanced NSCLC treated with PD-1 inhibitors	Better response and progression-free survival. These effects probably due to immune enhancement related to a prolonged retention of antigens on cell membrane and presentation increase [41]
	252 patients NSCLC, with 73 statin users (46 atorvastatin): hypercholesterolemia dosage	Evaluation of brain metastasis risk in lung cancer patients treated with statins: no significant results [97]
*Rosuvastatin*	Rosuvastatin (20–40 mg): high intensity	Better response and progression-free survival. These effects probably due to immune enhancement related to a prolonged retention of antigens on cell membrane and presentation increase [41]
*Simvastatin*	250 adenocarcinoma tissues (51 statin users) 5–10 mg Low-dose rosuvastatin, pitavastatin, fluvastatin and pravastatin were used, but with minor effects compared to simvastatin	Reduction of EMT, improved sensibility to EGFR-TKI and improved prognosis in adenocarcinoma patients holding p53 mutation. However, a worse outcome was described in wild-type p53 population. Survival of statin users was generally better [58]
	252 NSCLC patients, with 73 statin users (18 atorvastatin): hypercholesterolemia dosage	Evaluation of brain metastasis risk in lung cancer patients treated with statins: no significant results [97]
***Clinical trials***		
*Atorvastatin*	67 patients with advanced NSCLC (holding KRAS mutation): 20 mg	Better outcomes in those treated with EGFR-TKI plus simvastatin/atorvastatin than EGFR-TKI alone [98]
*Pravastatin*	The multicenter and randomized phase III trial LUNGSTAR: 846 SCLC patients: 40 mg	The first group received pravastatin plus chemotherapy (etoposide + cisplatin/carboplatin) vs. chemotherapy alone. No significant improvement in outcomes were reported [99]
*Simvastatin*	Phase II trial in 106 NSCLC patients: 40 mg.	No superiority of gefitinib + simvastatin compared to gefitinib alone. However, the combination therapy resulted in better RR and progression-free survival PFS in patients with EGFR adenocarcinomas (wild-type) [100]
	Phase 2 trial in 61 SCLC patients: 40 mg	Irinotecan + cisplatin + simvastatin showed no significant results [101] Ever-smokers showed a better overall survival compared to never smokers
	68 patients with non-adenocarcinomatous NSCLC (phase II): 40 mg	No significant results with simvastatin plus afatinib [102]
	67 patients with advanced NSCLC (holding KRAS mutation): 20 mg	Better outcomes in those treated with EGFR-TKI plus simvastatin/atorvastatin than EGFR-TKI alone [98]
***Metanalysis***		
*All statins*	A total of 23 studies were selected, including 15 observational studies and 8 RCTs. Various dosages, mainly hypercholesterolemia dosage	No protective effect of statins on lung cancer risk [103]
	Seventeen studies involving 98,445 patients. Various dosages, mainly hypercholesterolemia dosage	Decreased mortality in cohort studies, but not in clinical trials or case-control studies. Enhanced effect of EGFR-TKI [104]
	Twenty studies examined. Various dosages, mainly hypercholesterolemia dosage	No correlation between statin use and lung cancer risk [105]
	Nineteen studies involving 38,013 lung cancer patients. Various dosages, mainly hypercholesterolemia dosage	No correlation between statin use and lung cancer risk [106]

## Data Availability

No new data were created or analyzed in this study. Data sharing is not applicable to this article.

## Abbreviations

ABL	abelson tyrosine-protein kinase
ACLY	ATP citrate lyase
ACSL	Acyl-CoA synthetase long chain family member
ADP	adenosine diphosphate
Akt	protein kinase B
ALK	anaplastic lymphoma kinase
AMPK	5′ adenosine monophosphate-activated protein kinase
ApoB	apolipoprotein B
ARIC	atherosclerosis risk in communities
ATF	activation of transcription factor
AXL	anexelekto
BA	bioavailability
BAX	bcl-2-like protein 4
BCL	B-cell lymphoma (apoptosis gene)
Bcl-XL	B-cell lymphoma–extra large
BMI	body mass index
Cav	caveolin
CCI	Charlson comorbidity index
CCL	CC chemokine ligands
CCR	chemokine receptor type
CD	cluster of differentiation
CDC	cell division control
CDK	cyclin-dependent kinase
COPD	chronic obstructive pulmonary disease
COX	cyclooxygenase
CVD	cardiovascular disease
CYP450	cytochrome 450
CYR61	cysteine-rich angiogenic inducer 61
DNA	deoxyribonucleic acid
EGFR	epithelial growth factor receptor
EMT	Epithelial–mesenchymal transition
ERK	extracellular signal-regulated kinases
EZH	enhancer of zeste homolog
FADD	Fas-associated protein with death domain
FAS	fatty acid synthase
FPPS	farnesyl pyrophosphate synthase
FN	fibronectin
FOXP3	forkhead box P3
FTI	farnesyl transferase inhibitors
G_1_	Gap1
GDI	GDP-dissociation inhibitor
GGTI	geranylgeranyl transferase inhibitors
GLUT	glucose transporters
GTP	guanosine triphosphate
Gy	gray
H_2_O_2_	hydrogen peroxide
HDAC	histone deacetylase
hESC	human embryonic stem cells
HDL	high-density lipoprotein
HMG-CoA	hydroxy-3-methylglutaryl-*coenzyme A*
HNSCC	head and neck squamous cell carcinomas
HPV	human papillomavirus
HRT	hormone replacement therapy
hs-CRP	high sensitivity C-reactive protein
IAP	inhibitor of apoptosis
ICAM	intercellular adhesion molecule
ICB	immune checkpoint blockade
IC_50_	half maximal inhibitory concentration
IDOs	Indoleamine 2,3-dioxygenases
IL	interleukin
IR	ionizing radiation
IRF	interferon regulatory factor
KRAS	kirsten rat sarcoma virus
LDL	low density lipoprotein
LKB1	liver kinase B1
MAPK	mitogen-activated protein kinase
Mcl	myeloid leukemia cell differentiation protein
MCM	minichromosome maintenance
MDR	multidrug resistance
MEK	mitogen-activated protein kinase kinase
mESC	mouse embryonic stem cells
MET	mesenchymal epithelial transition tyrosine kinase
METTL3	methyltransferase 3
miRNA	micro-RNA
MMP	metalloproteinase
mRNA	messenger ribonucleic acid
MSC	mesenchymal stem cells
mTOR	mechanistic target of rapamycin
MyD88	myeloid differentiation primary response 88
MYC	myelocytomatosis (similar oncogene)
NADPH	nicotinamide adenine dinucleotide phosphate
NBP	nitrogen containing bisphosphonates
NFκB	nuclear factor kappa B
NGAL	neutrophil gelatinase-associated lipocalin
NNAL	nicotine-derived nitrosamine alcohol
NNK	nicotine-derives nitrosamine ketone
NOS	nitric oxide synthase
NSCLC	non-small cell lung cancer
OATP	organic anion transporting polypeptide
Oct4	octamer-binding transcription factor 4
PCOS	polycystic ovary syndrome
PDGFR	platelet-derived growth factor receptors
PD-1	programmed cell death protein 1
PD-L1	Programmed Death-Ligand 1
PFS	progression free survival
PI3K	phosphoinositide 3-kinases
PPAR-γ	peroxisome proliferator-activated receptors-gamma
PCCs	primary cancer cells
PTEN	phosphatase and tensin homolog
PTI	prenyltransferase inhibitors
p21	cyclin-dependent kinase inhibitor 1
p27	cyclin-dependent kinase inhibitor 1 B
Rac	ras-related C3 botulinum toxin substrate
RANKL	receptor activator of nuclear factor kappa-B ligand
RB	retinoblastoma protein
RCT	randomized controlled trial
RECK	reversion-inducing cysteine-rich protein with kazal motif
Rho	ras homologous protein
RIP	receptor interacting protein
ROS	receptor tyrosine kinase
RR	response rate
RT-PCR	real-time polymerase chain reaction
SCC	squamous cell lung carcinoma
SCLC	small cell lung cancer
SLCO	solute carrier organic anion transporter family member
SMA	smooth muscle actin
SMAD	small mother against decapentaplegic
SmPC	summary of product characteristics
SOX	SRY-related HMG-box genes
SREBP	sterol regulatory element-binding proteins
STAT	signal transducer and activator of transcription
TAZ	transcriptional coactivator with pdz-binding motif
TGF	transforming growth factor
TKI	tyrosine kinase inhibitor
TLR	Toll-like receptor
TNF	tumor necrosis factor
TRADD	tumor necrosis factor receptor type 1-associated death domain protein
TRAF	TNF-receptor associated factors
t_1/2_	half-life
UGT	uridine diphosphate-glucuronosyltransferase
Vi	vimentin
XIAP	X-linked inhibitor of apoptosis protein
YAP	yes-associated protein
VEGF	vascular endothelial growth factor
